# Tacrolimus (FK506) promotes placentation and maternal-fetal tolerance through modulating FASN-CEACAM1 pathway

**DOI:** 10.3389/fimmu.2025.1522346

**Published:** 2025-02-19

**Authors:** Xinhang Meng, Minfeng Shi, Jinfeng Qian, Yujie Luo, Liyuan Cui, Dajin Li, Songcun Wang

**Affiliations:** ^1^ Laboratory for Reproductive Immunology, Hospital of Obstetrics and Gynecology, Fudan University Shanghai Medical College, Shanghai, China; ^2^ Reproductive Medicine Center, Changhai Hospital, Naval Medical University, Shanghai, China

**Keywords:** recurrent pregnancy loss, tacrolimus (FK506), placentation, HTR8/SVneo cells, maternal-fetal tolerance

## Abstract

The establishment of placentation and maternal-fetal tolerance are important determinants of a successful pregnancy. Tacrolimus, also known as FK506, is a calcineurin inhibitor that has often been used for pregnant women after solid organ transplantation. Previous therapeutic interventions have shown the benefits of using the immuno-suppressive agent FK506 in improving clinical pregnancy and live birth rates and reducing the risk of spontaneous miscarriage. However, the mechanism(s) by which FK506 is involved in these processes have not been fully elucidated. To further characterize its function in early pregnancy, we explored the effect of FK506 on the human-derived first trimester extravillous trophoblast cells (HTR8/SVneo cells) and found that FK506 promoted invasion, tube formation and proliferation, but inhibited apoptosis of HTR8/SVneo cells. Based on the integrated metabolomics and transcriptomics analyses, the present study provided the cellular and molecular cues evidently showing that FK506 had positive effects on the placentation and maternal-fetal tolerance through modulating FASN-CEACAM1 pathway. The spontaneous-abortion-prone model gave further evidence that FK506 exerted a protective effect on pregnancy by regulating the FASN-CEACAM1 axis. These findings might provide a new fundamental mechanism and promising potential of low-dose FK506 in preventing pregnancy loss.

## 1 Introduction

Pregnancy loss, defined as the spontaneous end of a pregnancy before the fetus reaches viability, is a common female reproductive disease. The prevalence of pregnancy loss increases with maternal age and even rises to 50% in women over 40 years old (1). Recurrent pregnancy loss (RPL, two or more consecutive miscarriages) account for 1%-5% of pregnant women (2, 3). RPL profoundly affects the quality of life of women and their partners. It can be argued that the physical and psychological burden on women is compounded with each pregnancy loss they experience. In addition, women with a history of RPL are at a higher risk of subsequent pregnancy loss (4). Accordingly, it is imperative to explore the underlying mechanisms of RPL and identify its therapeutic targets.

Although several risk factors have been associated with RPL (2, 3), including chromosomal abnormalities, uterine anatomic abnormalities, endocrine abnormalities, metabolic abnormalities, infections and immune disorders, etc., approximately 50% of RPL causes are still unexplained (1). During pregnancy, human extravillous trophoblast cells (EVTs) invade into the maternal decidua and uterine blood vessels, dissolve the extracellular matrix, remodel the uterine vasculature and come into direct contact with the maternal decidual immune cells (5). Development of the allogeneic fetus in the maternal uterus requires the maternal immune system to accept the fetus expressing allogeneic paternal antigens as well as to provide competent responses to infections. Thus, the establishment of successful placentation and maternal-fetal tolerance are the basis of a successful pregnancy (6). Inadequate placental development and impaired induction of maternal-fetal tolerance have been found to be closely related to RPL (6, 7).

Tacrolimus, also known as FK506, was found as a 23-membered macrolide produced by *Streptomyces*. The complex of FK506 and its intracellular binding protein negatively affect the cytoplasmic nuclear factor of the activated T cell pathway by inhibiting calcineurin and interleukin (IL) -2 transcription, thereby inhibiting the production of IL-6, IL-1β and tumor necrosis factor (TNF) -α, and the proliferation of T cell-dependent B cells, resulting in a powerful immunosuppressive effect. Calcineurin inhibitors are widely used as immunosuppressants in solid organ transplant recipients and patients with autoimmune disorders. FK506 has been defined by the Food and Drug Administration as a Class C drug for pregnancy (8). FK506, in particular, has often been used for pregnant women after solid organ transplantation (9). FK506 could also have a potential efficacy against obstetrical complications associated with immune bias disorders, such as RPL, recurrent implantation failure (10–12).

Recently, FK506 was also reported to promote the migration and invasion of the human-derived first trimester extravillous trophoblast cells (HTR8/SVneo cells), suggesting an immune-independent action mode of FK506 in positively influencing placentation *in vitro (* 13). To explore the mechanism by which FK506 functions on biological behaviors of EVTs, in the present study, FK506-treated HTR8/SVneo cells were collected and analyzed by transcriptomics and metabolomics. The involvement of FK506 in the maintenance of pregnancy and development of the placenta was also explored in a spontaneous-abortion-prone (SA) model.

## 2 Results

### 2.1 FK506 promoted the migration, invasion, proliferation and tube formation in HTR8/SVneo cells

**Figure 1:**
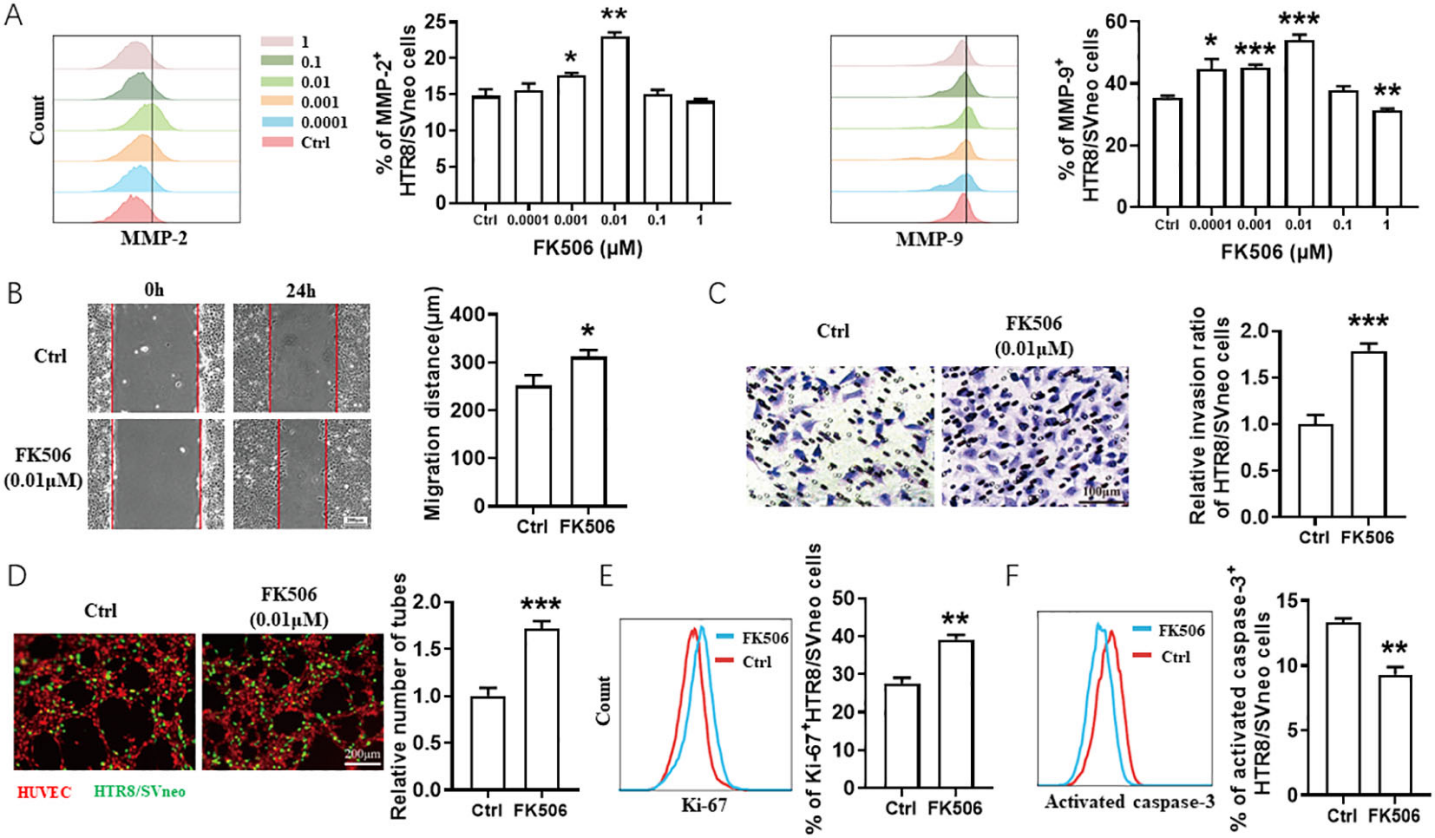
FK506 promoted the migration, invasion, proliferation and tube formation in HTR8/SVneo cells. **(A)** Flow cytometric analysis and quantitation of MMP-2 and MMP-9 expression on HTR8/SVneo cells treated with a range of concentrations of FK506 (0, 0.0001, 0.001, 0.01, 0.1, and 1 µM) for 48 hours. Ctrl, DMSO. **(B)** The wound-healing assays of HTR8/SVneo cells after treated with DMSO or 0.01 µM FK506 for 48 hours. Representative images obtained along the wounds at 0 and 24 hours. **(C)** Matrigel invasion assays of HTR8/SVneo cells after treated with DMSO or 0.01 µM FK506 for 48 hours. **(D)** Tube formation assay of HUVECs (red) and HTR8/SVneo cells (green) after treated with DMSO or 0.01 µM FK506 for 48 hours. Images are representative of three individual experiments. **(E)** Flow cytometric analysis and quantitation of Ki-67 expression on HTR8/SVneo cells treated with DMSO or 0.01 µM FK506 for 48 hours. **(F)** Flow cytometric analysis assessing the activated caspase-3 of HTR8/SVneo cells treated with DMSO or 0.01 µM FK506 for 48 hours. Data represent the mean ± SEM and are representative of three independent analyses. *p<0.05, **p<0.01, ***p<0.001.

To investigate the potential role of FK506 in placenta development, we studied the effect of FK506 on the HTR8/SVneo cell biological behaviors, including migration, invasion, tube forming ability, proliferation and apoptosis. As shown in **Figure 1A**, with the concentration range of 0.0001-1 μM, both 0.001 and 0.01 μM FK506 upregulated matrix metalloproteinase (MMP)-2 and MMP-9 expression of HTR8/SVneo cells, which reached a peak in the FK506 concentration of 0.01 μM. Thus, we used the concentration 0.01 μM in the follow-up experiments.

The gap of the wound was significantly reduced by migrating HTR8/SVneo cells after treated with 0.01 µM FK506 for 24 hours (**Figure 1B**). The numbers of HTR8/SVneo cells penetrating through the polycarbonate membranes coated with Matrigel and the tube formation by human umbilical vein endothelial cells (HUVECs) and HTR8/SVneo cells co-culture system increased under the treatment of FK506 (**Figures 1C, D**). FK506 also promoted proliferation (assessed by the Ki-67, **Figure 1E**), but inhibited apoptosis (according to the proportion of activated caspase-3^+^ cells, **Figure 1F**) of HTR8/SVneo cells. These observations support previously published data indicating that FK506 has the potential to regulate biological behaviors of HTR8/SVneo cells and thus might play an important role in placentation (13, 14).

### 2.2 FK506 promoted the migration, invasion, proliferation and tube formation in HTR8/SVneo cells via inhibiting fatty acid synthase (FASN)

**Figure 2:**
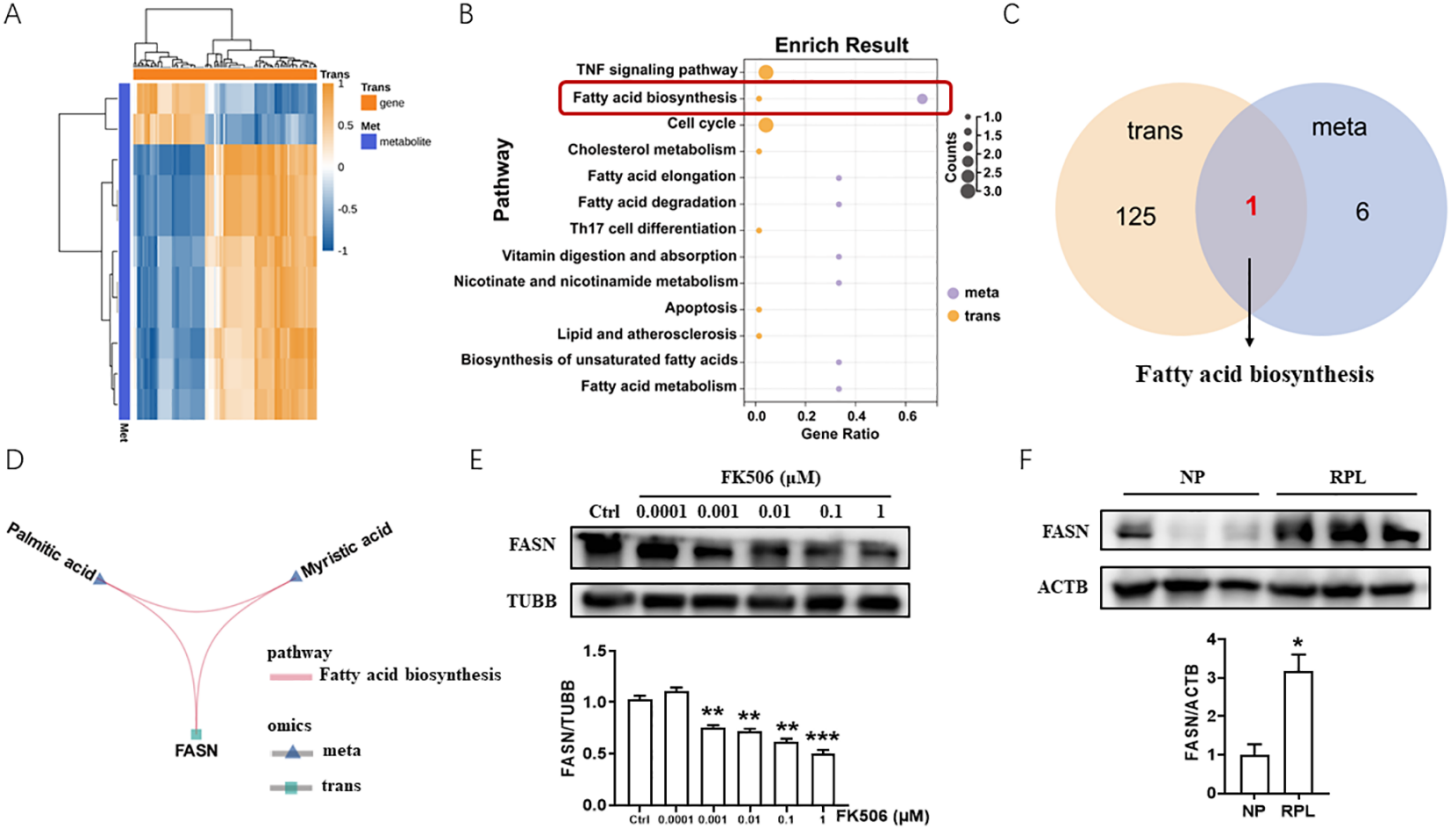
Integrated analysis of transcriptomics and metabolomics of FK506-treated HTR8/SVneo cells. **(A)** The top 100 differentially expressed genes and their correlations with the differential metabolites between 0.01 μM FK506 exposure and control group (DMSO). **(B)** KEGG analysis of differentially expressed genes and differential metabolites caused by FK506 treatment of HTR8/SVneo cells. **(C)** Differentially expressed genes and differential metabolites caused by FK506 were significantly enriched in pathways of fatty acid biosynthesis. **(D)** Among the differentially expressed genes and differential metabolites caused by FK506, one key gene (*FASN*) and two metabolites (palmitic acid and myristic acid) were mapped simultaneously into the fatty acid biosynthesis pathway. **(E)** The protein level of FASN in HTR8/SVneo cells treated with a range of concentrations of FK506 for 48 hours were examined by Western blot. Ctrl, DMSO. β-tubulin (TUBB) was used as an internal control. **(F)** The protein level of FASN in the placental villus from NP and RPL examined by Western blot. Images are representative of three individual experiments. β-actin (ACTB) was used as an internal control. Data represent the mean ± SEM and are representative of three independent analyses. *p<0.05, **p<0.01, ***p<0.001.

To explore the mechanism of FK506 function on the biological behaviors of EVTs, FK506-treated HTR8/SVneo cells were collected and detected by transcriptomics and metabolomics. Afterwards integrated analysis of differentially expressed genes and metabolites between FK506 exposure groups and control groups were conducted to systematically reveal the molecules and signaling pathways associated with the response to FK506 exposure. The top 100 differentially expressed genes and their correlations with the differential metabolites are shown in **Figure 2A**. To better understand the relationship between genes and metabolites, differentially expressed genes and differential metabolites caused by FK506 treatment of HTR8/SVneo cells were mapped to the Kyoto Encyclopedia of Genes and Genomes (KEGG) pathway map. The results indicated that these genes and metabolites were significantly enriched in pathways of fatty acid biosynthesis (**Figures 2B, C**). One key gene (*FASN*) and two metabolites (palmitic acid and myristic acid) were mapped simultaneously into the fatty acid biosynthesis pathway (**Figure 2D**). In addition, FK506 inhibited FASN expression in HTR8/SVneo cells (**Figure 2E**). The expression of FASN also increased in the villus of RPL patients (**Figure 2F**), we speculated that FK506 might promote the migration, invasion, proliferation and tube formation in HTR8/SVneo cells via inhibiting FASN.

**Figure 3:**
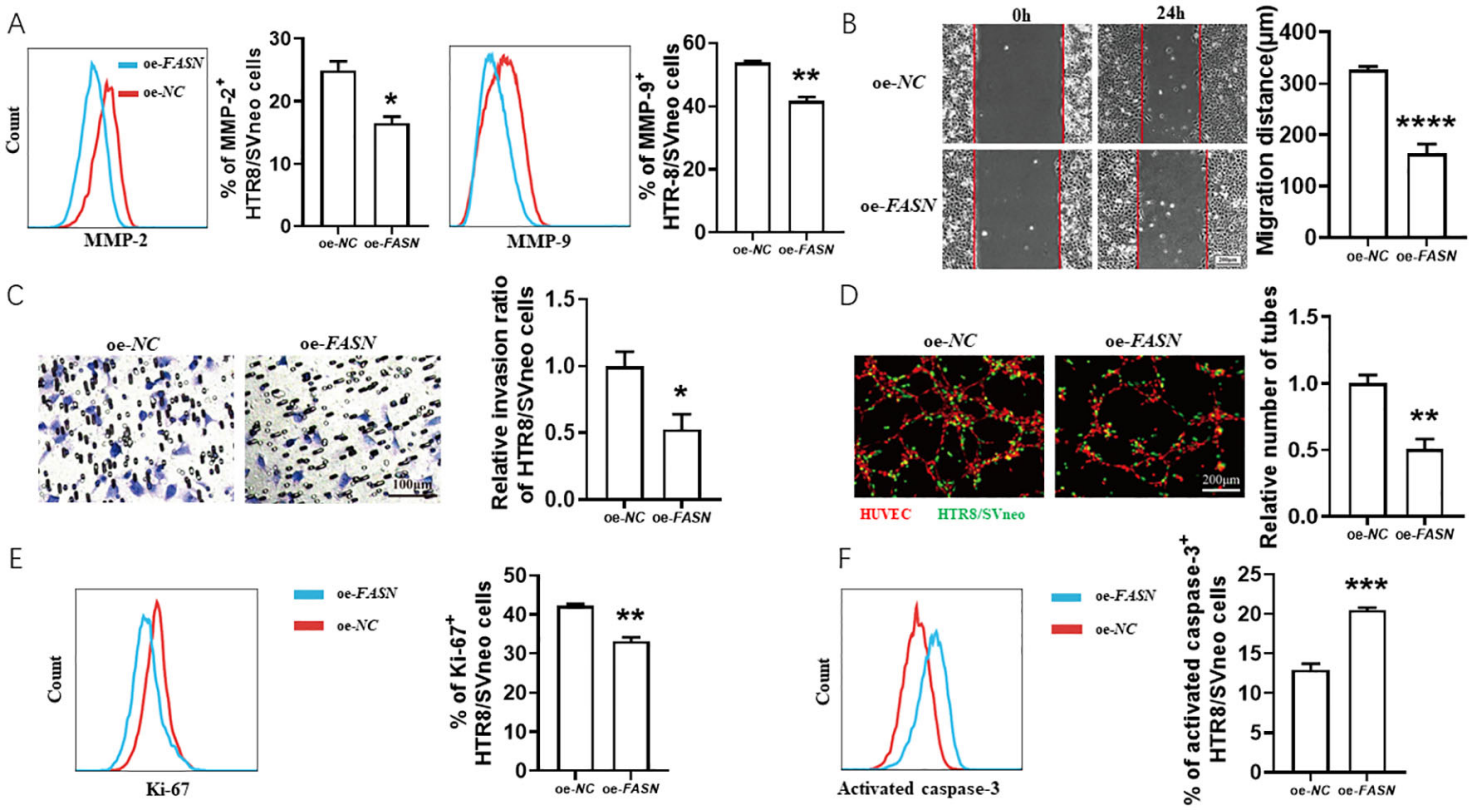
FK506 promoted the migration, invasion, proliferation and tube formation in HTR8/SVneo cells via inhibiting FASN. **(A)** Flow cytometric analysis and quantitation of MMP-2 and MMP-9 expression in HTR8/SVneo cells after transfected with empty vector or *FASN* overexpression plasmid in the exposure of 0.01 μM FK506 for 48 hours. oe, overexpression. *NC*, negative control. **(B–D)** The wound-healing assay **(B)**, Matrigel invasion assay **(C)** of HTR8/SVneo cells and tube formation assay of HUVECs (red) and HTR8/SVneo cells (green) co-culture system **(D)** with the indicated treatments. **(E, F)** Flow cytometric analysis and quantitation of Ki-67 **(E)** and activated caspase-3 **(F)** expression of HTR8/SVneo cells transfected with empty vector or FASN overexpression plasmid in the exposure of 0.01 μM FK506 for 48 hours. Images are representatives of three independent experiments. Flow cytometry plot is from one representative experiment. Data represent mean ± SEM. *p<0.05, **p<0.01, ***p<0.001, ****p<0.0001.

As shown in **Figure 3**, *FASN* overexpression decreased FK506-induced MMP-2 and MMP-9 expression (**Figure 3A**) in HTR8/SVneo cells, inhibited the FK506-induced HTR8/SVneo cell migration (**Figure 3B**) and invasion (**Figure 3C**), reduced the tube formation of HUVECs and HTR8/SVneo cells co-culture system (**Figure 3D**), and also reversed the regulation of FK506 on the proliferation (**Figure 3E**) and apoptosis (**Figure 3F**) of HTR8/SVneo cells.

### 2.3 Carcinoembryonic antigen-related cell adhesion molecule 1 (CEACAM1) rescued the adverse effects of *FASN* overexpression on HTR8/SVneo cells

**Figure 4:**
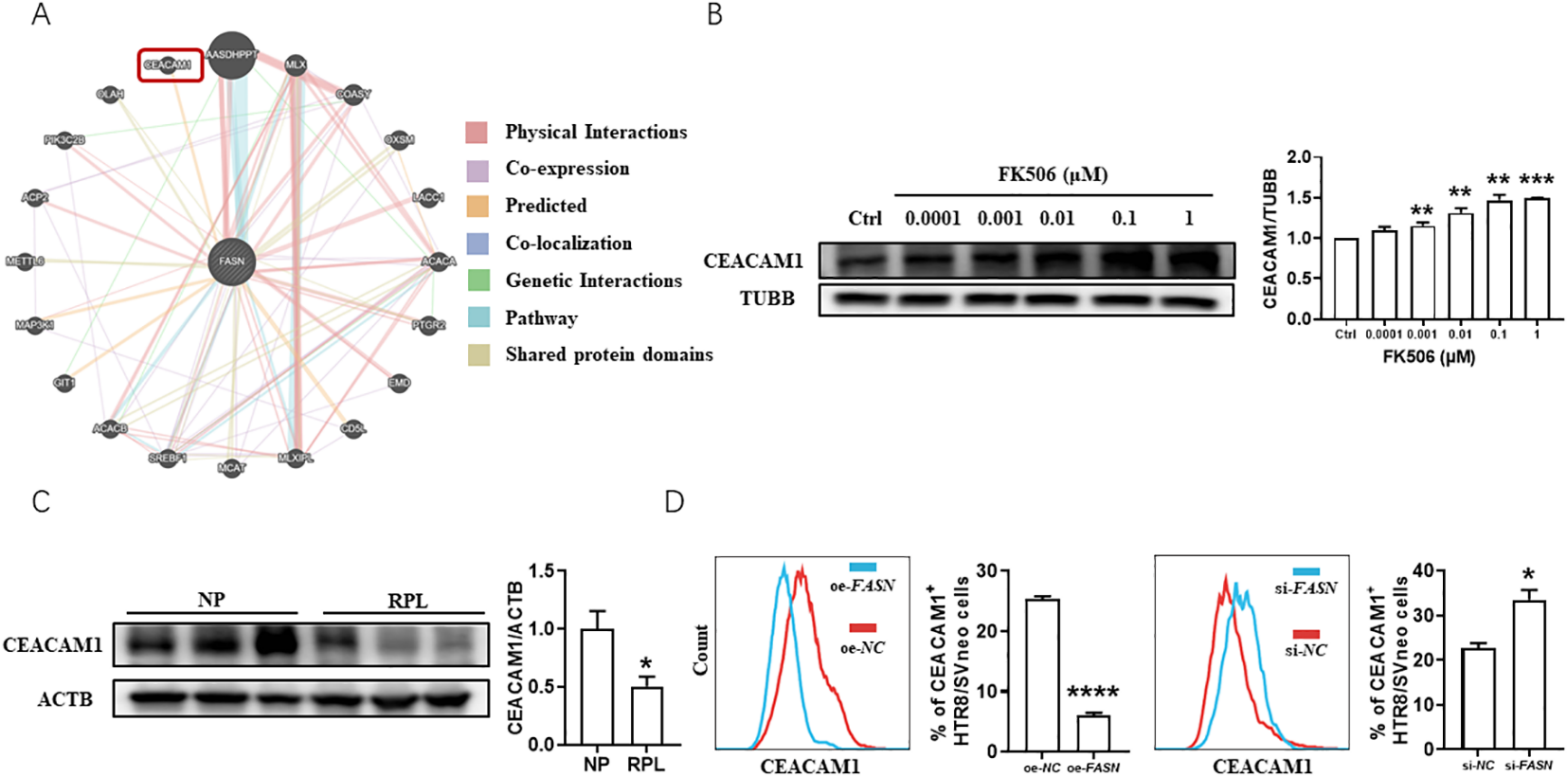
Evidence of a potential reciprocal interaction between FASN and CEACAM1 in the placenta of RPL patients and in HTR8/SVneo cells. **(A)** GeneMANIA Cytoscape program predicted that *FASN* might interact with *CEACAM1.* **(B)** The protein level of CEACAM1 in HTR8/SVneo cells treated with a range of concentrations of FK506 for 48 hours were examined by Western blot. β-tubulin (TUBB) was used as an internal control. **(C)** The protein level of CEACAM1 in the placental villus from NP and RPL examined by Western blot. β-actin (ACTB) was used as an internal control. **(D)** Flow cytometric analysis and quantitation of CEACAM1 expression of HTR8/SVneo cells after transfected with *FASN* overexpression plasmid or *FASN*-specific siRNA for 48h. *NC*, negative control. oe, overexpression. si, small interfering RNA. Images are representatives of three independent experiments. Flow cytometry plot is from one representative experiment. Data represent mean ± SEM. *p<0.05, **p<0.01, ***p<0.001, ****p<0.0001.

Gene-gene interaction studies, as analyzed using GeneMANIA Cytoscape program v.3.6.0 (https://genemania.org/) (15), demonstrated that *FASN* might interact with *CEACAM1* (**Figure 4A**), which serves important roles in trophoblast cell invasion (16) and immune response control (17). This attracted our interests as trophoblast cell invasion and maternal-fetal tolerance are the key aspects of successful pregnancy. FK506 increased CEACAM1 expression in HTR8/SVneo cells (**Figure 4B**). Compared to clinically normal first trimester pregnancies, the expression of CEACAM1 decreased in the villus of RPL patients (**Figure 4C**). CEACAM1 blockade reduced FK506-induced MMP-2 and MMP-9 expression (**Supplementary Figure 2A**) and also reversed the regulation of FK506 on the proliferation (**Supplementary Figure 2B**) and apoptosis (**Supplementary Figure 2C**) of HTR8/SVneo cells. In addition, *FASN* overexpression inhibited CEACAM1 expression, while *FASN* downregulation promoted CEACAM1 expression in HTR8/SVneo cells (**Figure 4D**, see also **Supplementary Figures 1A, B**).

**Figure 5:**
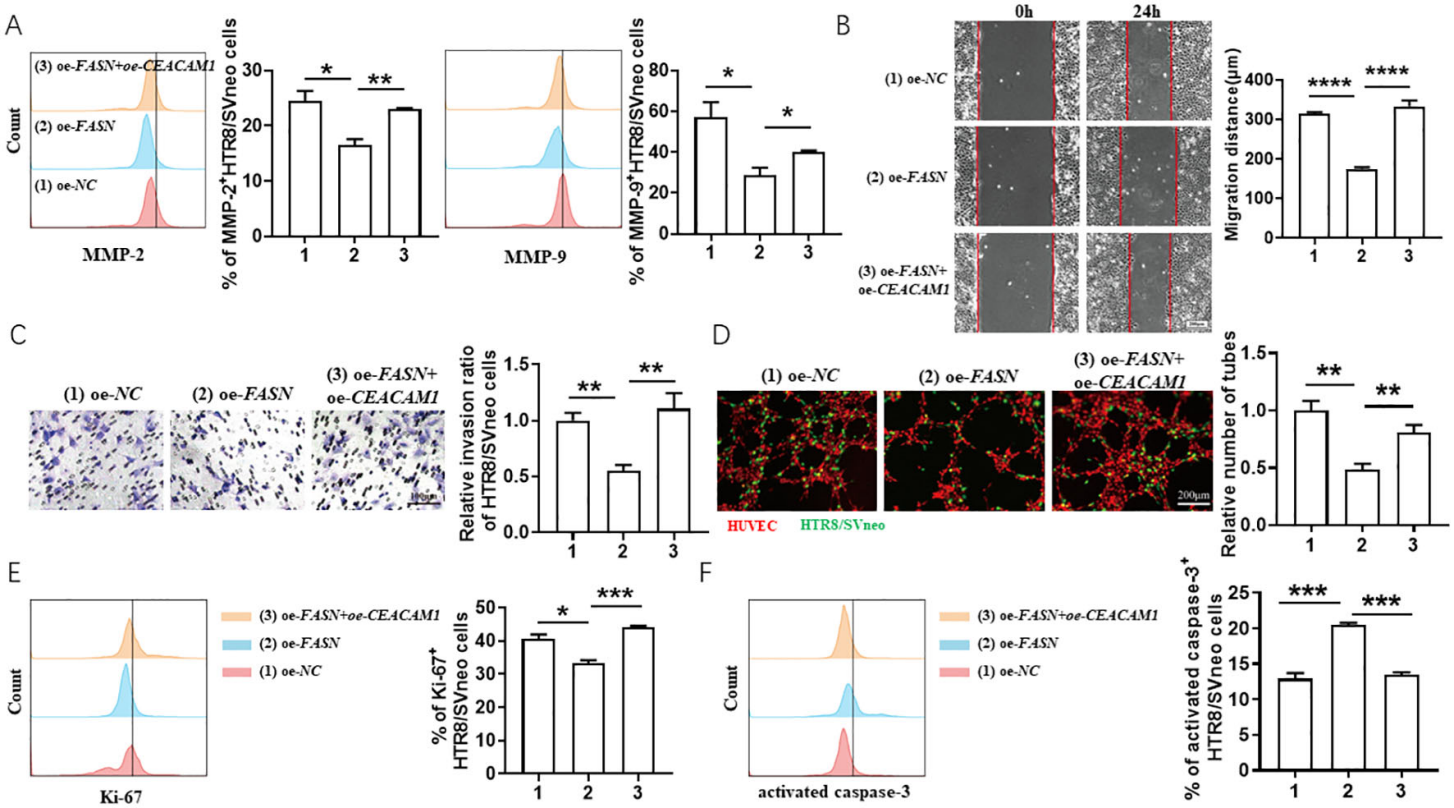
*CEACAM1* overexpression rescued the adverse effects of *FASN* overexpression on HTR8/SVneo cells. **(A)** Flow cytometric analysis and quantitation of MMP-2 and MMP-9 expression in HTR8/SVneo cells after transfected with empty vector, *FASN* overexpression plasmid or co-transfected with *FASN* overexpression plasmid and *CEACAM1* overexpression plasmid in the exposure of 0.01 μM FK506 for 48 hours. **(B–D)** The wound-healing assays **(B)**, Matrigel invasion assays **(C)** of HTR8/SVneo cells and tube formation assay of HUVECs and HTR8/SVneo cells coculture system **(D)** with the indicated treatments. **(E, F)** Flow cytometric analysis and quantitation of Ki-67 **(E)** and activated caspase-3 **(F)** expression on HTR8/SVneo cells with the indicated treatments. Images are representatives of three independent experiments. Flow cytometry plot is from one representative experiment. *NC*, negative control, oe, overexpression. Data represent mean ± SEM. *p<0.05, **p<0.01, ***p< 0.001, ****p< 0.0001.

With the intention of revealing the regulatory relationship between CEACAM1 and FASN, co-transfection of CEACAM1 and FASN in HTR8/SVneo cells was conducted. *CEACAM1* overexpression rescued the adverse effects of *FASN* overexpression on HTR8/SVneo cells. As shown in **Figure 5**, under the exposure of 0.01 μM FK506, *CEACAM1* overexpression reversed *FASN*-overexpression-induced MMP-2 and MMP-9 downregulation (**Figure 5A**) in HTR8/SVneo cells, promoted migration (**Figure 5B**) and invasion ability (**Figure 5C**) of HTR8/SVneo cells inhibited by *FASN* overexpression, increased the tube formation of HUVECs and *FASN*-transfected HTR8/SVneo cells co-culture system (**Figure 5D**), as well as reversed the regulation of *FASN* overexpression on the proliferation (**Figure 5E**) and apoptosis (**Figure 5F**) of HTR8/SVneo cells. Hence, there was a close regulatory relationship between CEACAM1 and FASN, and the trade-offs between them profoundly affected the biological behaviors of HTR8/SVneo cells.

### 2.4 FK506 alleviated fetal loss in SA model in a FASN-CEACAM1 dependent way

**Figure 6:**
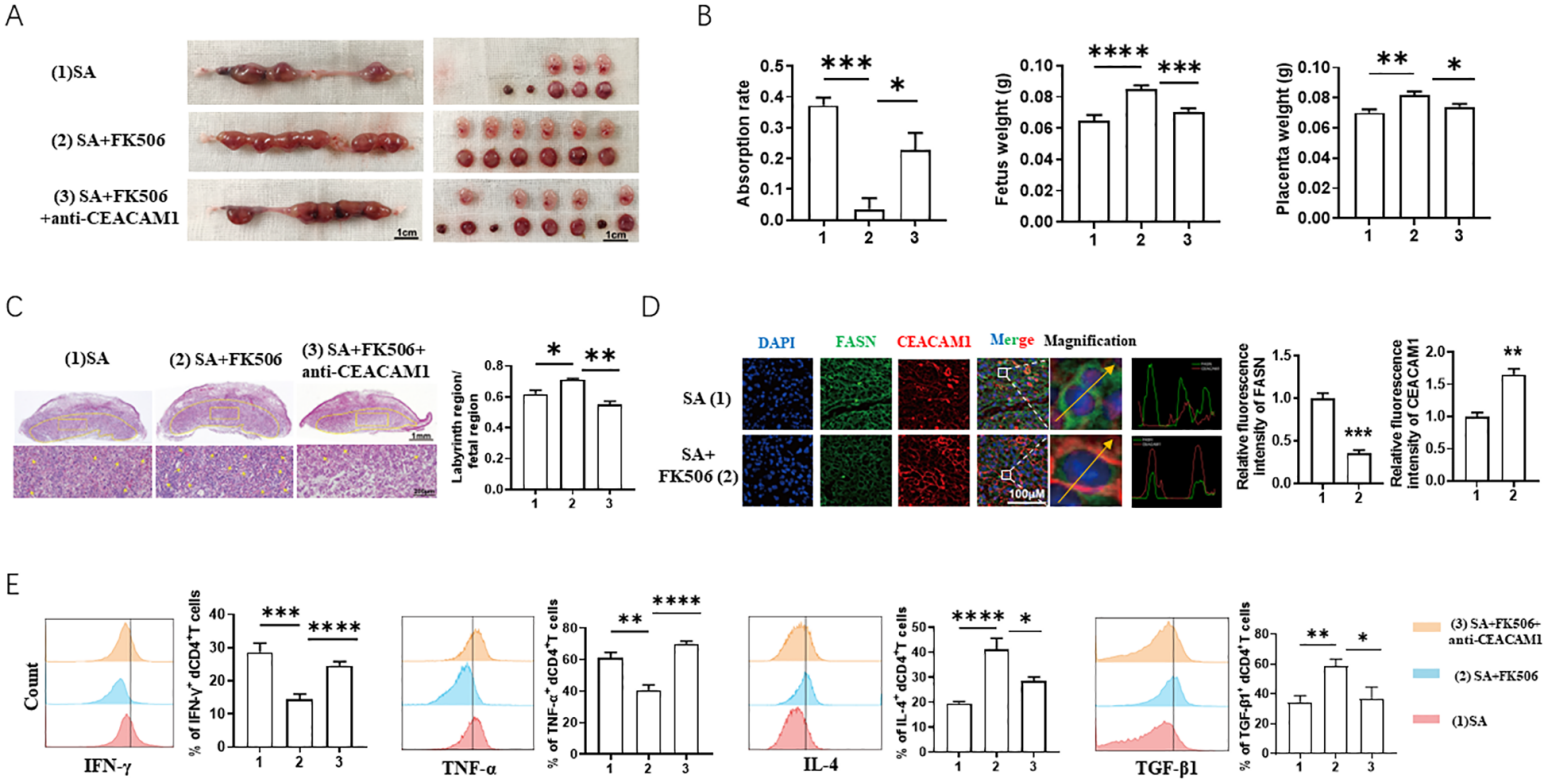
FK506 alleviated fetal loss of SA models in a FASN-CEACAM1 dependent way. **(A, B)** Representative images of uterus **(A)**, statistics of absorption rate, fetal weight and placental weight **(B)** of SA mice with the indicated treatments. **(C)** Representative images of placental hemi-sections (H&E-stained) of SA mice with the indicated treatments. **(D)** Immunofluorescence analysis and quantitation of placental FASN and CEACAM1 expression of SA mice with the indicated treatments. **(E)** Flow cytometric analysis and quantitation of IFN-γ, TNF-α, IL-4 and TGF-β1 expression on decidual CD4^+^T cells of SA mice with the indicated treatments. Flow cytometry plot and images are representative of three individual experiments. Data represent mean ± SEM. *p<0.05, **p<0.01, ***p< 0.001, ****p< 0.0001.

To further verify the therapeutic effect and mechanism of FK506 in pregnancy loss, we established a SA model (female CBA/J mice mated with male DBA/2 mice). FK506 intraperitoneal injection decreased the absorption rate of SA mice (**Figure 6A, B**), along with improvement of labyrinth region in placentae (**Figure 6C**), downregulation of FASN and upregulation of CEACAM1 (**Figure 6D**). However, the protection effects of FK506 were resisted by additional anti-CEACAM1 antibody treatment, which is reflected by the higher absorption rate and poorer development of labyrinth region in placentae compared with FK506 alone treatment (**Figures 6A–C**). In addition, the flow cytometry analysis revealed increased expression of Th2 and Treg cytokines (IL-4, transforming growth factor (TGF) -β1) but decreased expression of Th1 cytokines (TNF-α, interferon (IFN) -γ) in decidual CD4^+^T cells in SA mice after FK506 treatment. Nonetheless, the Th2 and Treg bias in the pregnant uterus induced by FK506 was abrogated after CEACAM1 blockade (**Figure 6E**). Thus, blocking CEACAM1 counteracted the alleviation of adverse pregnancy outcome of SA mice by FK506.

## 3 Discussion

**Figure 7:**
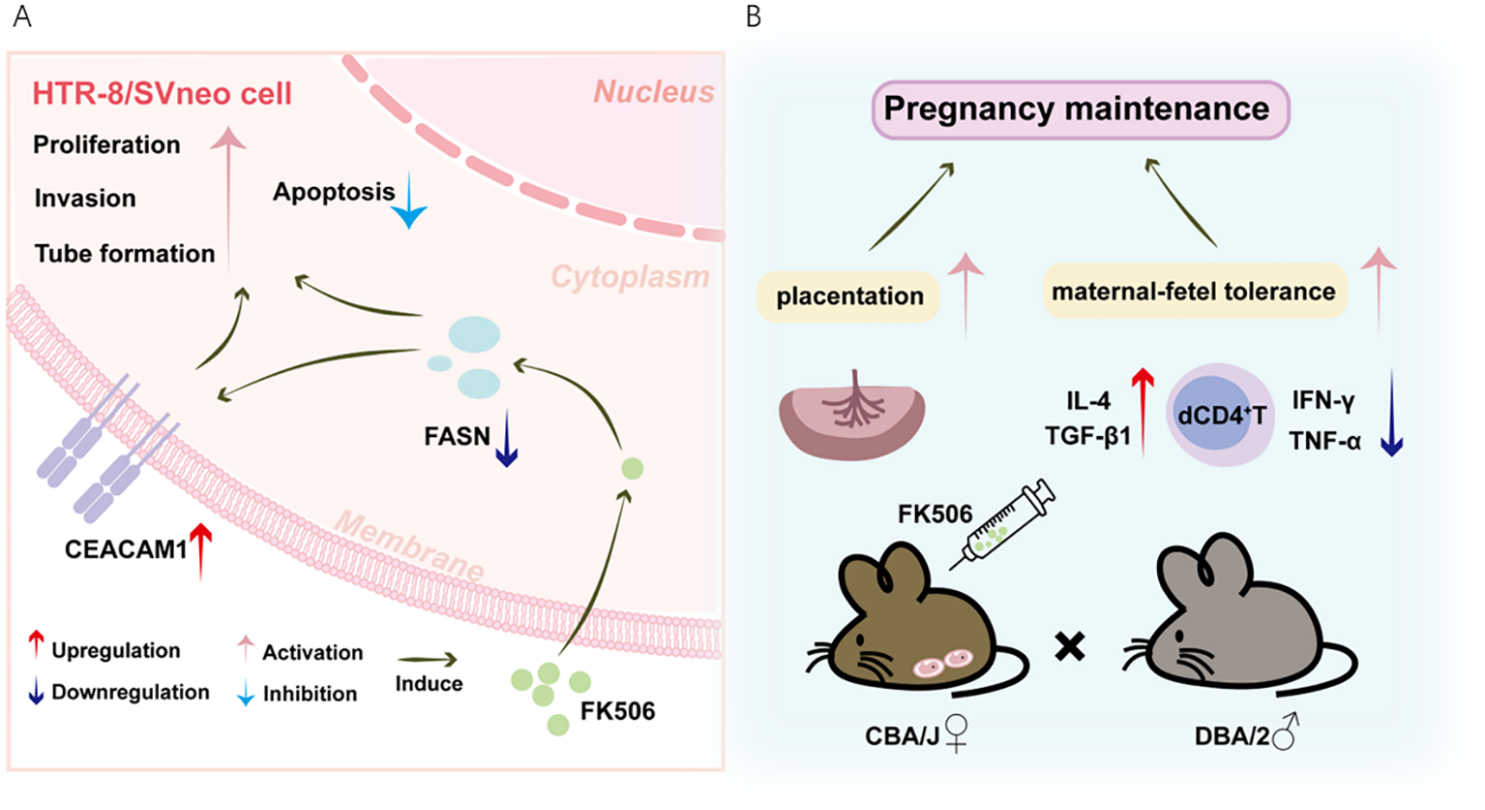
Schematic diagram of FK506-mediated positive effects on the placentation and maternal-fetal tolerance through modulating FASN-CEACAM1 pathway. **(A)** FK506 promoted invasion, tube formation and proliferation, but inhibited apoptosis of HTR8/SVneo cells. Based on the integrated metabolomics and transcriptomics analyses, FK506 might regulate biological behaviors of HTR8/SVneo cells via inhibiting FASN, which might interact with CEACAM1. FK506 increased CEACAM1 expression on HTR8/SVneo cells. In turn, CEACAM1 rescued the adverse effects of FASN on HTR8/SVneo cells. **(B)** The SA mouse model gave further evidence that FK506 exerted a protective effect on pregnancy by promoting placentation and inducing Th2/Treg bias in the pregnant uterus to maintain maternal-fetal tolerance through regulating the FASN-CEACAM1 axis.

The embryo carries both maternal and paternal antigens, thus the development of the allogeneic fetus in the maternal uterus represents an allograft. Previous therapeutic interventions have shown the benefits of using the immuno-suppressive agent FK506 in mitigating the incidence of implantation failure and RPL, preventing placentation defects, and restoring proper spiral artery remodeling (10–12, 18, 19). However, the mechanism(s) by which FK506 is involved in these processes is yet to be fully comprehended. Based on the integrated metabolomics and transcriptomics analyses, the present study provided further cellular and molecular cues evidently demonstrating that FK506 can positively influence placentation and promote maternal-fetal tolerance at least in part through modulating FASN-CEACAM1 pathway (**Figure 7**).

Our studies on the FK506-induced promotion of migration, invasion, tube formation and proliferation, as well as inhibition of apoptosis of HTR8/SVneo cells supported recently reported immune-independent mechanism of FK506 in promoting the functional capacities of trophoblast cells during pregnancy *in vitro (* 14). The improved maternal-fetal vascular network in the labyrinth of FK506-treated SA mice gave further insights that FK506 could positively influence placentation. The combined analysis of transcriptome and metabolome supported with Western blot indicated that FASN, a multifunctional enzyme necessary for the *de novo* synthesis of long-chain fatty acids (20), might be a downstream target of FK506 in influencing placentation.

The role of FASN during RPL remains poorly understood. Increased expression of FASN in male sperm of RPL couples has been reported (21, 22). Furthermore, placental *FASN* expression related negatively to placental weight (23). In the present study, we revealed that the expression of FASN was significantly increased in RPL villus. FK506 downregulated FASN expression in HTR8/SVneo cells, and functionally, *FASN* overexpression suppressed the migration, invasion and tube formation capacities of HTR8/SVneo cells induced by FK506. These data gave further clues that the relatively low expression of FASN might be important for the maintenance of a healthy pregnancy, as there was a potential correlation between high levels of FASN and RPL. However, FK506 could induce dyslipidemia by upregulating FASN during liver transplantation (24). This seems to be contrary to our findings and may be due to the higher dosage of FK506 used during organ transplantation (25), further reminding us to use low-dose FK506 during pregnancy and pay attention to its effective concentration range and adverse reactions. As placental *FASN* expression also related positively to circulating FASN, studies to address changes in FASN in plasma would be informative and might have more clinical value that FASN might act as a potential biomarker for RPL diagnosis and therapy (23).

Gene-gene interaction studies predicted that *FASN* might interact with *CEACAM1.* CEACAM1 is a member of the immunoglobulin as the ligand of T-cell immunoglobulin mucin-3 (Tim-3) (26). CEACAM1 not only suppresses the inflammatory response but also initiates extracellular matrix remodeling during tumor development (27). Notably, CEACAM1 also plays important roles in trophoblast cell invasion (16). It has been reported that CEACAM1 could interact with FASN to promote hepatic insulin clearance so as to maintain normo-insulinemia and insulin sensitivity (28, 29). We also found that there might be a colocalization between FASN and CEACAM1 in placenta (**Figure 6D**). Nevertheless, the molecular mechanisms between them need to be further investigated. In the present study, CEACAM1 expression was decreased in RPL villus, and *CEACAM1* overexpression could rescue the adverse effects of *FASN* overexpression on HTR8/SVneo cells. Thus, the relative higher CEACAM1 expression might be beneficial to the maintenance of pregnancy. CEACAM1 expression was downregulated in diabetes mellitus and high-fat diet mouse model, therefore, controlling diet to maintain glucose and lipid metabolism balance might be also a potential therapeutic strategy in RPL (30). FK506 upregulated CEACAM1 expression on HTR8/SVneo cells and placenta, and anti-CEACAM1 canceled the protective effect of FK506 on HTR8/SVneo cells, as well as placental development and maternal-fetal tolerance of SA mice, suggesting that FK506 exerted a protective effect on pregnancy by regulating the FASN-CEACAM1 axis.

Though HTR8/SVneo cells have been proven effective for recapitulating key aspects of EVTs (31), questions remain regarding the validity of using immortalized cell lines to represent the *in vivo* environment. Data obtained in the present study further extend the notion that the underlying mechanisms of FK506 capacity for regulating trophoblast functions and maternal-fetal tolerance are associated with FASN-CEACAM1 axis, highlighting the promising potential of low-dose FK506 (0.01 μM in the present study) in preventing pregnancy loss. Nonetheless, the functional regulation of FK506 on primary trophoblasts and the related mechanisms still require further study.

## 4 Materials and methods

### 4.1 Human samples

The use and collection of human samples were approved by the Human Research Ethics Committee of the Obstetrics and Gynecology Hospital of Fudan University (No. Kyy2021-11). All participants provided written informed consent. All the methods were carried out in accordance with the approved guidelines. Human villi from human first-trimester pregnancies were obtained from RPL patients (RPL group) and clinically normal pregnancies (NP group, terminated for non-medical reasons, had at least one successful pregnancy and no history of spontaneous abortions). Subjects with RPL included those undergoing spontaneous abortion and who also had a history of two or more consecutive spontaneous abortions without known causes (including parental or embryonic karyotype anomalies, uterine anatomic abnormalities, infection-associated factors, endocrine disorders and antiphospholipid syndrome, etc.). None of the subjects had any history of autoimmune diseases or immunotherapy, hormone therapy, renal or liver diseases, alcohol addiction, smoking or vaccination within 3 months before sample collection.

### 4.2 Cell treatment

HTR8/SVneo cells were grown in DMEM/F12 medium supplemented with 10% fetal bovine serum (FBS), 100 U/mL penicillin, 100 μg/mL streptomycin, and 1 μg/mL amphotericin B at 37°C in 5% CO_2_. HTR8/SVneo cells were cultured 12 hours in complete medium and further incubated in serum-free medium for 12 hours, followed by treatment with a range of concentrations of FK506 (F138016, Aladdin, China, dissolved in Dimethyl sulfoxide (DMSO), 0, 0.0001, 0.001, 0.01, 0.1, and 1 µM) for 48 hours. In some experiments, HTR8/SVneo cells were dealt with anti-human CEACAM1 neutralizing antibody (Biocampare, ABIN5684089), *FASN*-specific small interfering RNA (siRNA) (si-*FASN*, 5’-GGAGCGUAUCUGUGAGAAAT-3’, 5’-UUUCUCACAGAUACGCUCCTT-3’), *FASN* overexpression plasmid and/or *CEACAM1* overexpression plasmid (Public Protein/Plasmid Library, PPL, China) in the exposure of 0.01 µM FK506 for 48 hours using transfection reagent (L3000015, Thermo Fisher Scientific, U.S.A or Polyethylenimine Linear (PEI) MW40000 (49553-93-7, Yeasen Biotechnology, Shanghai, China), according to the manufacturer’s instructions. Notably, 0.01 μM is the optimal concentration of FK506 to promote the invasion in HTR8/SVneo cells, which is equivalent to 8.2203 ng/mL (as the molar mass of Tacrolimus Monohydrate is 822.03) and close to 10 ng/mL in the previous publications (13, 14).

### 4.3 Western blot

The tissue and cell samples were lysed with cold radio-immunoprecipitation buffer (Beyotime Biotechnology, China) supplemented with a protease inhibitor cocktail (C0001, TargetMol, China). Protein concentrations were determined by the BCA Protein Assay Kit (WB6501, ncmbiotech, China). Lysates were heated at 95°C for 10 minutes, and then loaded on 7.5% gels (Bio-Rad, U.S.A) for SDS-polyacrylamide gel electrophoresis. After electrophoretic separation, the proteins were transferred onto 0.45 μm PVDF membranes (ipvh00010, Millipore, Germany), blocked with 5% defatted milk, and incubated overnight at 4°C with the primary antibodies (anti-FASN (ab128870, Abcam, U.S.A), anti-CEACAM1 (ab300061, Abcam, U.S.A), anti-CEACAM1(283340, Invitrogen, U.S.A), anti-β-Tubulin (ab6046, Abcam, U.S.A) and anti-Actin (ab179467, Abcam, U.S.A)). Membranes were washed and incubated with Horseradish peroxidase (HRP)-conjugated secondary antibody (Jackson, U.S.A) at room temperature for 1 hour. The antibody-labeled proteins were detected by chemiluminescence instrument (Amersham™ Imager 600, GE Healthcare, U.S.A) using Chemiluminescent Kit (Share-bio, China).

### 4.4 Wound-healing assay

HTR8/SVneo cell migration was measured by determining the ability of the cells to move into an acellular space, as described previously (32). Briefly, the linear scratches were made when HTR8/SVneo cells with indicated treatment were grown to 90% confluence. Then, the cells were gently washed with phosphate-buffered saline (PBS) for three times to remove medium with FK506. The cells were allowed to grow in serum-free medium, and the width of the wound was monitored at the indicated time of wound healing after treatment under the phase-contrast microscope. Photographs were taken and the relative distance traveled by the cells at the acellular front was measured at 5 different marked crossed locations per well.

### 4.5 Matrigel invasion assay

Cell culture inserts were precoated with Matrigel (Corning, U.S.A.) and placed in a 24-well plate. HTR8/SVneo cells (2 × 10^4^ in 200 μL of serum-free medium) pre-treated with FK506, specific siRNA or overexpression plasmid for 48 hours were plated in the upper chamber. The lower chamber was filled with 500 μL of DMEM/F12 with 10% FBS. The cells were incubated at 37°C for 48 hours. After the inserts fixed with 4% paraformaldehyde (PFA) (G1101, Servicebio, China) and stained with crystal violet solution (G1063, Solarbio, China), the Matrigel and non-invading cells were removed from the upper surface of the filter by wiping with a cotton swab gently. Cells were observed using an Olympus BX51tDP70 fluorescence microscope (Olympus, Japan). Cells that had migrated to the lower surface were counted at a magnification of 100×. Each experiment was carried out in triplicate and repeated three times independently.

### 4.6 Tube formation assay

*In vitro* cell tube formation was assayed by determining the ability of the cells to form tubes on a 3D Matrigel scaffold. Matrigel (356234, BD Biosciences, San Jose, CA, USA) was plated into 96‐well plates at 50 μL per well and polymerized for 30 minutes at 37 °C. HUVECs stained with cell tracker red fluorescent probe (C2925, Invitrogen, CA, USA) were plated at 2 × 10^4^ cells per well and HTR8/SVneo cells (pre-treated with FK506, specific siRNA or overexpression plasmid for 48 hours as described earlier), which were stained with cell tracker green fluorescent probe (C2927, Invitrogen, CA, USA), were seeded at 2 × 10^4^ cells per well in 100 μL DMEM/F12 with 10% FBS. Tube formation was visualized under a fluorescence microscope after 4 hours.

### 4.7 Combined metabolomics and transcriptomics analysis

HTR8/SVneo cells treated with DMSO or 0.01 µM FK506 for 48 hours were set up for transcriptomics and metabolomics sequencing. The number of sequenced reads falling in the exons of this meta-gene was calculated using feature Counts tool in subread, and the differential expression analysis was conducted by DE-Seq. P < 0.001 were regarded as the significance threshold. Differential genes were enriched by the KEGG in gene set enrichment analysis. The metabolites with the variable importance in the projection≥1 and P-value of Student’s t-test ≤0.05 were considered as significantly changed. The KEGG database was used to annotate and display the differential metabolites. Other analyses included partial least- squares discriminant analysis and pathway enrichment.

### 4.8 Mice

CBA/J female and DBA/2 male mice were purchased from Slac laboratory animal Co. (Shanghai, China) and Huafukang bioscience Co. (Beijing, China) and maintained in an animal facility according to institutional and National Institutes of Health Guidelines. Eight-week-old CBA/J females were mated to DBA/2 males to establish SA models (33). All the CBA/J females were inspected every morning for vaginal plugs. The day of visualization of a plug was designated as day 0.5 of pregnancy. Pregnant mice were divided into 3 groups: SA group, SA group receiving injections of FK506 on day 4.5, SA group receiving injections of FK506 (0.05 mg/kg) on day 4.5 and anti-CEACAM1 antibody (Clone: MAb-CC1, 134534, Biolegend, U.S.A) at doses of 250 μg, 125 μg, and 125 μg on days 4.5, 6.5, and 8.5, respectively. All pregnant mice were monitored at day 13.5 of pregnancy. The percentage of fetal loss (the embryo absorption rate) was calculated as following: % of absorption = R/(R + V) × 100, where R represents the number of hemorrhagic implantation (sites of fetal loss) and V stands for the number of viable, surviving fetuses.

### 4.9 Preparation of mouse cells

Uteri from pregnant mice were dissected free from the mesometrium and removed by cuts at the ovaries and cervix. The fetal and placental tissues were carefully removed and washed in PBS. Minced uteri were digested in RPMI 1640 medium supplemented with collagenase type IV and DNase I for 45 minutes at 37°C with gentle agitation. Cells were cultured in RPMI 1640 medium supplemented with 10% FBS, 100 U/mL penicillin, 100 μg/mL streptomycin and 1 μg/mL amphotericin B at 37°C in 5% CO_2_ for 4 hours to remove adherent stromal cells. Anti-mouse CD3 (5 μg/mL, Clone: 145-2C11, 100340, Biolegend, U.S.A), anti-mouse CD28 (1 μg/mL, Clone: 37.51,102116, Biolegend, U.S.A), phorbol 12-myrstate 13-acetate (50 ng/mL, Biolegend, U.S.A.), ionomycin (1 μg/mL, Biolegend, U.S.A.) and brefeldin A (10 mg/mL, BioLegend, U.S.A.) were added 4 hours for intracellular cytokine analysis.

### 4.10 Hematoxylin and eosin (H&E) staining of placental hemi-sections

Mouse placentae were fixed in 4% PFA overnight and embedded in paraffin. Serial 3 µm thick mid-sagittal sections were cut and used for H&E staining. Labyrinth vascular areas were observed using an Olympus BX51tDP70 fluorescence microscope (Olympus, Japan). In all cases, the central region of the placenta was examined. An average of five nonconsecutive sagittal sections at an interval of at least 40 µm for each placenta was used for the measurement.

### 4.11 Immunofluorescence staining

Mouse placentae were fixed in 4% PFA overnight and embedded in paraffin. Serial 3 µm thick mid-sagittal sections were cut and used for immunofluorescence staining. The tissue sections were dewaxed and rehydrated in xylene and ethanol gradients. Then, the slides were immersed in citric acid antigen repair buffer (pH 6.0, G1219-1L, Servicebio, China) and heated at 98 °C for 15 minutes for antigen retrieval. After cooling to room temperature, the sections were blocked with 10% donkey serum (SL050, solarbio, China) for 1 hour at room temperature before incubation with primary antibodies (anti-FASN (ab128870, Abcam, 1:400), anti-CEACAM1(283340, Invitrogen, U.S.A, 1:200), anti-CEACAM1 (A25475, ABclonal, China, 1:100)) overnight at 4 °C. The next day, the slides were placed in PBS and washed three times on a decolorizing shaker before incubation with Alexa Fluor-conjugated secondary antibodies (AS027, AS039, AS053, AS054, ABclonal, China, 1:100) for 1 hour at room temperature. The sections were subsequently counterstained with 4′,6-diamidino-2-phenylindole (DAPI, G1012, Servicebio, China) and mounted with anti-fade mounting medium. Images were captured on a THUNDER Imaging Systems (Leica, Germany). Mean fluorescence intensities was determined by Image J software and the relative fluorescence intensities of FASN and CEACAM1 in the placentae was calculated as following: Relative fluorescence intensity = X/S, where S represents the mean fluorescence intensities of all cells of three fields of view of the placentae from SA mice, and X stands for the mean fluorescence intensities of cells in each field of view of the placentae from SA mice and SA mice with FK506 administration. The fluorescence curves represented the fluorescence density value along the direction of the arrow.

### 4.12 Flow cytometry

Cell surface molecular expression and intracellular cytokine production were evaluated using flow cytometry. AlexaeFluor^®^ 488-conjugated anti-mouse IL-4 (Biolegend, U.S.A); APC-conjugated anti-human MMP-9 (Biolegend, U.S.A); PE-conjugated anti-human MMP-2 (R&D, U.S.A) or anti-human active caspase-3 (BD Biosciences, U.S.A); PE/CY7-conjugated anti-mouse TNF-α (Biolegend, U.S.A); PE-TexasRed-conjugated anti-mouse IFN-γ; Brilliant Violet 421-conjugated anti-human Ki-67 or anti-mouse TGF-β1; Brilliant Violet 510-conjugated anti-mouse CD4 (Biolegend, U.S.A). For intracellular staining, cells were fixed and permeabilized using the Fix/Perm kit (Biolegend, U.S.A). Isotype antibodies were used for setting gates. Flow cytometry was performed on a Beckman-Coulter CyAn ADP cytometer and analyzed with FlowJo software (Tree Star, U.S.A.).

### 4.13 Statistical analysis

All variables were normally distributed in this study. Thus, variables were presented as means and standard error of mean (SEM). Significance of differences between two groups was determined by Student’s *t*-test. Multiple groups were analyzed by one-way ANOVA with Bonferroni posttests or the *post-hoc* Dunnett *t*-test using Prism Version 8 software (GraphPad, San Diego, CA, USA). For all statistical tests, *p*- values <0.05 were considered statistically significant.

## Data Availability

The original contributions presented in the study are included in the article/**Supplementary Material**. Further inquiries can be directed to the corresponding authors.

## Supplementary material

Supplementary Figure 1 The protein level of FASN and CEACAM1 in HTR8/SVneo cells after indicated treatment examined by Western blot. β-actin (ACTB) was used as an internal control.

Supplementary Figure 2
**(A)** Flow cytometric analysis and quantitation of MMP-2 and MMP-9 expression in HTR8/SVneo cells after CEACAM1 blockade in the exposure of 0.01 μM FK506 for 48 hours. **(B, C)** Flow cytometric analysis and quantitation of Ki-67 **(B)** and activated caspase-3 **(C)** expression on HTR8/SVneo cells with the indicated treatments. Images are representatives of three independent experiments. Flow cytometry plot is from one representative experiment. Ctrl, DMSO. Data represent mean ± SEM. **p*<0.05, ***p*<0.01, ****p*< 0.001.
