# Supplementary figures and images for: Tacrolimus (FK506) promotes placentation and maternal-fetal tolerance through modulating FASN-CEACAM1 pathway
Source: Front Immunol. 2025 Feb 19;16:1522346. doi: 10.3389/fimmu.2025.1522346 (PMC11879939; doi:10.3389/fimmu.2025.1522346)

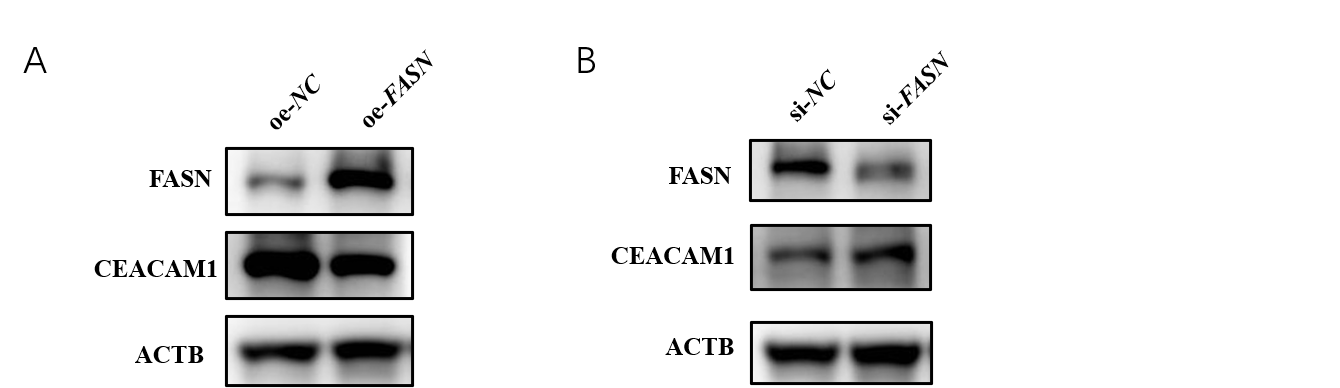

Supplement: Supplementary Figure 1 — The protein level of FASN and CEACAM1 in HTR8/SVneo cells after indicated treatment examined by Western blot. β-actin (ACTB) was used as an internal control. [file Image1.tif]

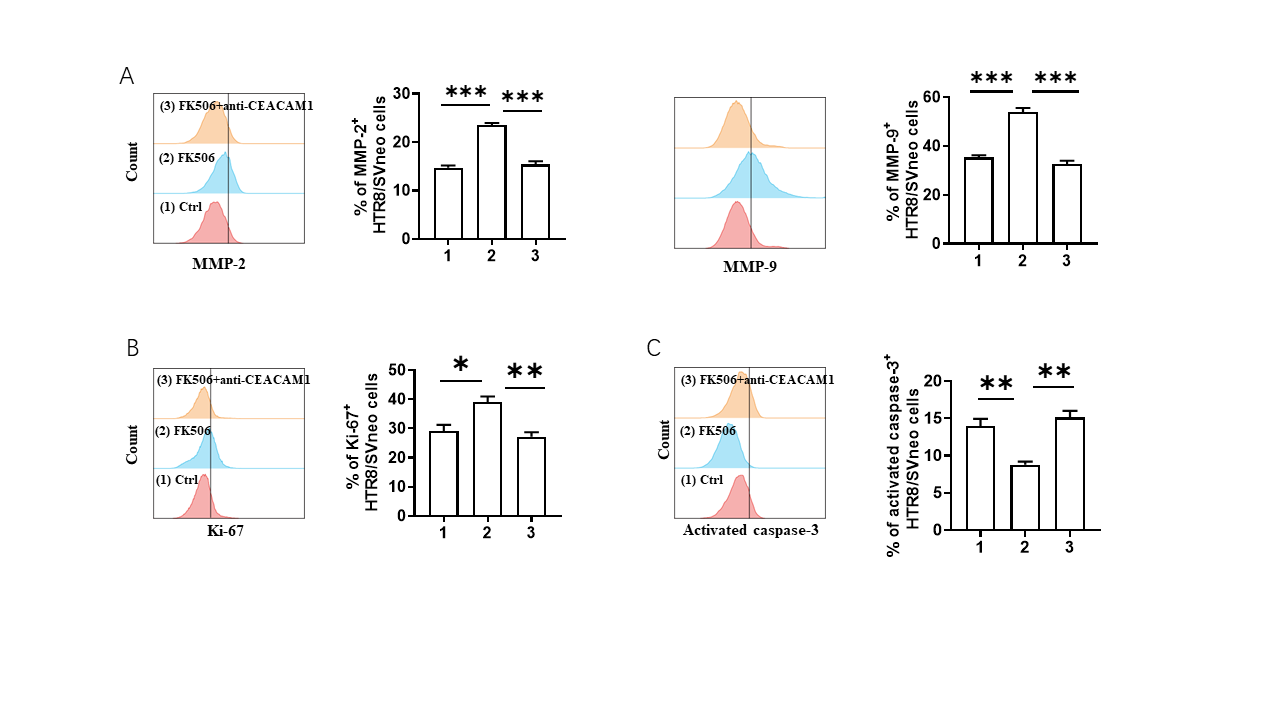

Supplement: Supplementary Figure 2 — (A) Flow cytometric analysis and quantitation of MMP-2 and MMP-9 expression in HTR8/SVneo cells after CEACAM1 blockade in the exposure of 0.01 μM FK506 for 48 hours. (B, C) Flow cytometric analysis and quantitation of Ki-67 (B) and activated caspase-3 (C) expression on HTR8/SVneo cells with the indicated treatments. Images are representatives of three independent experiments. Flow cytometry plot is from one representative experiment. Ctrl, DMSO. Data represent mean ± SEM. *p<0.05, **p<0.01, ***p< 0.001. [file Image2.tif]
