# Supplementary material for: Tacrolimus (FK506) promotes placentation and maternal-fetal tolerance through modulating FASN-CEACAM1 pathway
Source: Front Immunol. 2025 Feb 19;16:1522346. doi: 10.3389/fimmu.2025.1522346 (PMC11879939; doi:10.3389/fimmu.2025.1522346)
Supplement: Supplementary file 3 [file Presentation1.pptx]

## Slide 1
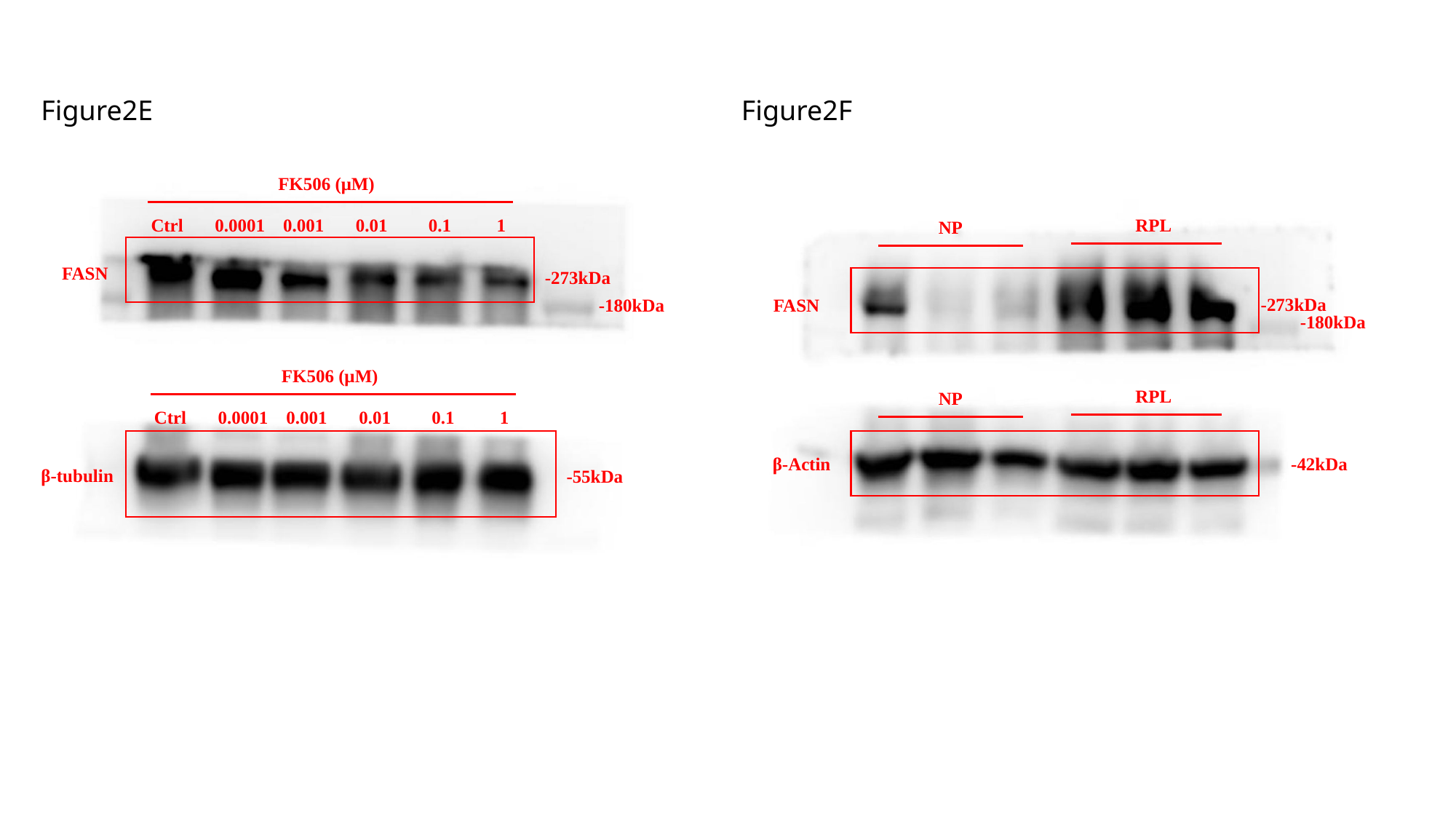

Figure2E
Figure2F
FK506 (μM)
RPL
 Ctrl 0.0001 0.001 0.01 0.1 1
NP
FASN
-273kDa
-273kDa
-180kDa
FASN
-180kDa
FK506 (μM)
RPL
NP
 Ctrl 0.0001 0.001 0.01 0.1 1
β-Actin
-42kDa
β-tubulin
-55kDa

## Slide 2
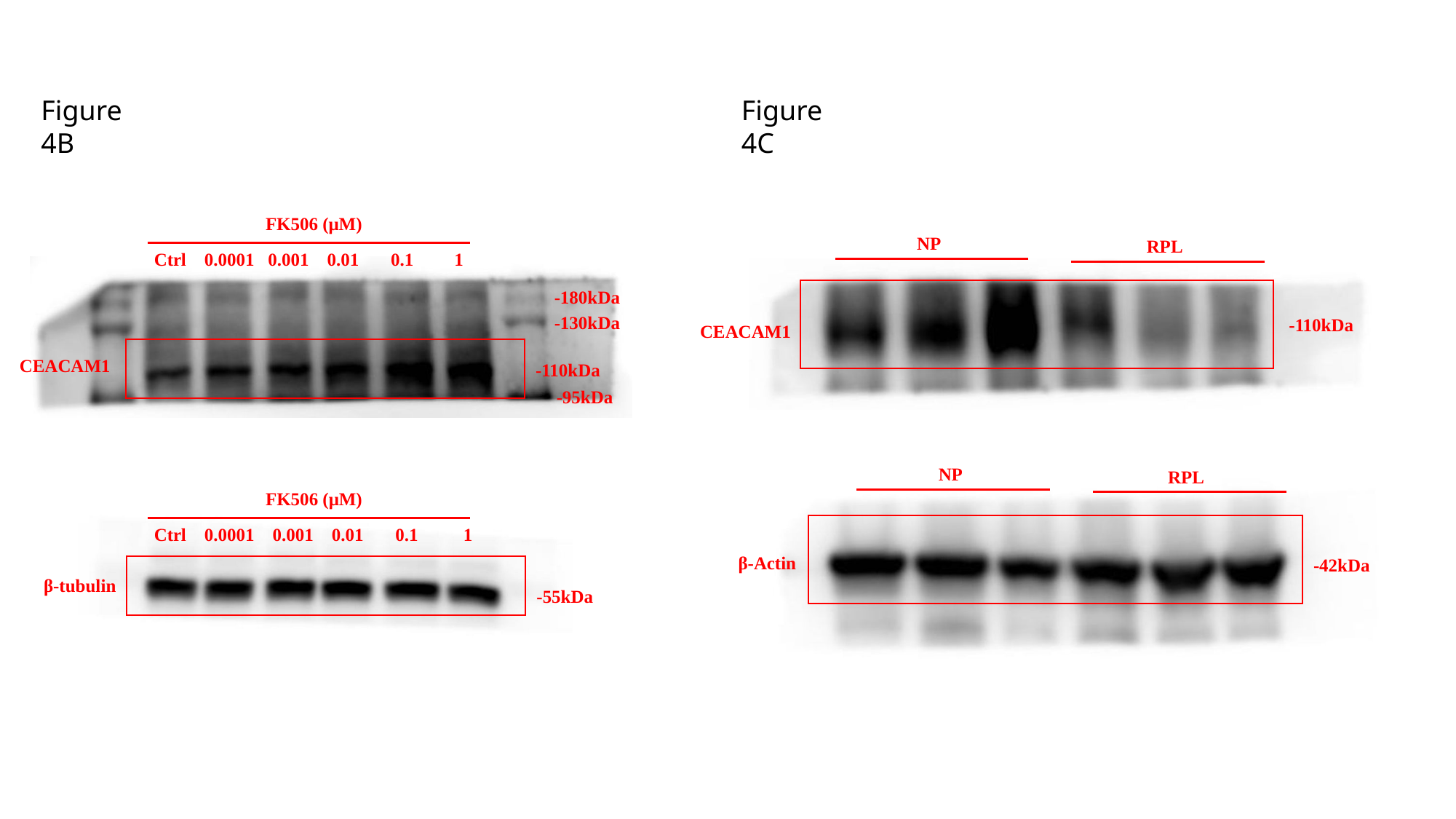

Figure 4B
Figure 4C
FK506 (μM)
NP
RPL
 Ctrl 0.0001 0.001 0.01 0.1 1
-180kDa
-130kDa
-110kDa
CEACAM1
CEACAM1
-110kDa
-95kDa
NP
RPL
FK506 (μM)
 Ctrl 0.0001 0.001 0.01 0.1 1
β-Actin
-42kDa
β-tubulin
-55kDa

## Slide 3
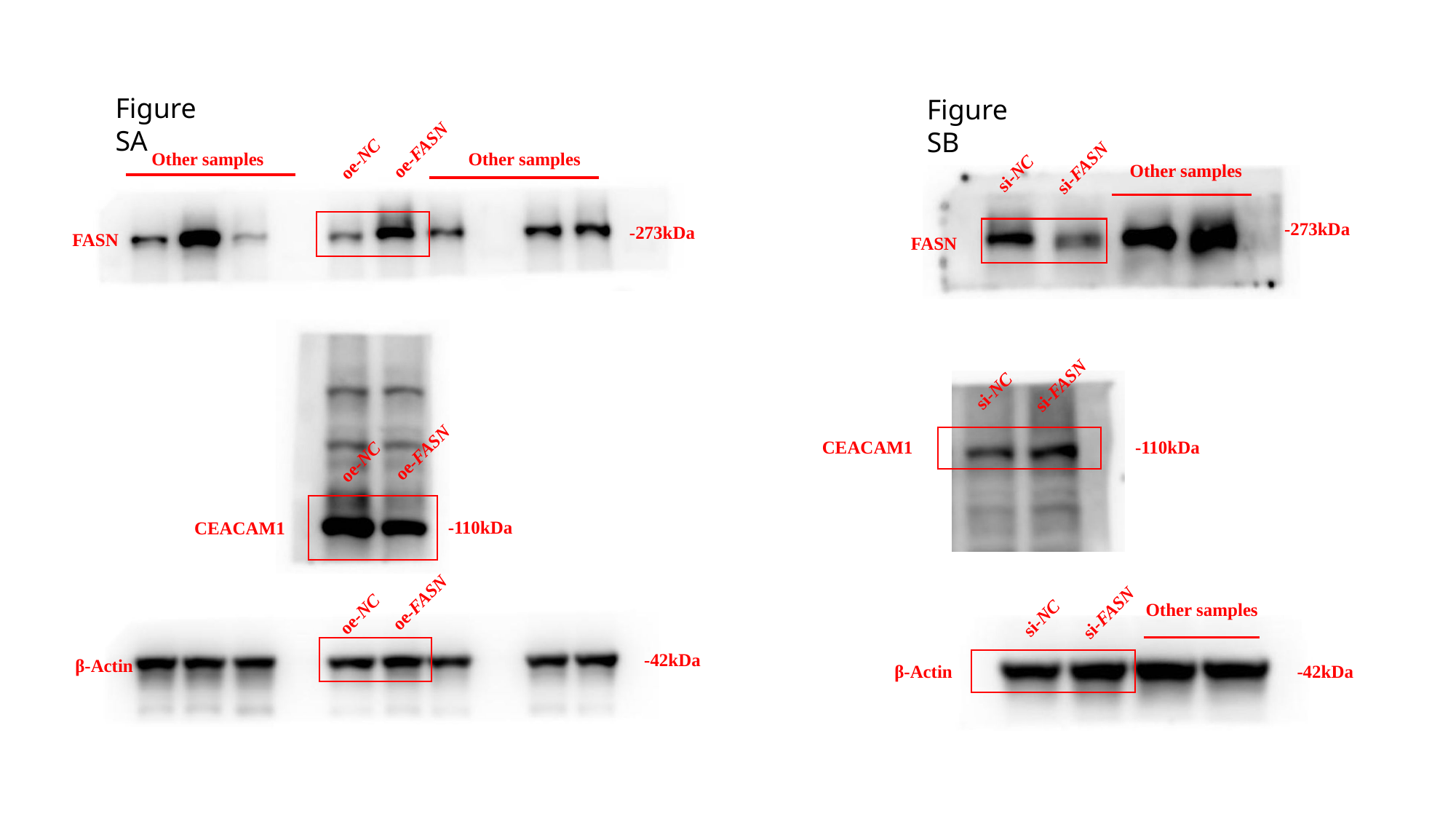

Figure SA
Figure SB
oe-FASN
oe-NC
si-FASN
si-NC
Other samples
Other samples
Other samples
-273kDa
-273kDa
FASN
FASN
si-FASN
si-NC
oe-FASN
-110kDa
CEACAM1
oe-NC
-110kDa
CEACAM1
oe-FASN
oe-NC
si-FASN
si-NC
Other samples
-42kDa
β-Actin
-42kDa
β-Actin
